# Genome‐wide time‐to‐event analysis on smoking progression stages in a family‐based study

**DOI:** 10.1002/brb3.462

**Published:** 2016-04-22

**Authors:** Liang He, Janne Pitkäniemi, Kauko Heikkilä, Yi‐Ling Chou, Pamela A.F. Madden, Tellervo Korhonen, Antti‐Pekka Sarin, Samuli Ripatti, Jaakko Kaprio, Anu Loukola

**Affiliations:** ^1^ Department of Public HealthUniversity of HelsinkiHelsinkiFinland; ^2^ Finnish Cancer RegistryInstitute for Statistical and Epidemiological Cancer ResearchHelsinkiFinland; ^3^ Institute for Molecular Medicine Finland (FIMM)University of HelsinkiHelsinkiFinland; ^4^ Washington University School of MedicineDepartment of PsychiatrySt. LouisMissouri; ^5^ National Institute for Health and WelfareHelsinkiFinland; ^6^ Institute of Public Health and Clinical NutritionUniversity of Eastern FinlandKuopioFinland; ^7^ Wellcome Trust Sanger InstituteHinxtonCambridgeUK

**Keywords:** Cessation, genome‐wide association study, initiation, smoking behavior, time‐to‐event analysis

## Abstract

**Background:**

Various pivotal stages in smoking behavior can be identified, including initiation, conversion from experimenting to established use, development of tolerance, and cessation. Previous studies have shown high heritability for age of smoking initiation and cessation; however, time‐to‐event genome‐wide association studies aiming to identify underpinning genes that accelerate or delay these transitions are missing to date.

**Methods:**

We investigated which single nucleotide polymorphisms (SNPs) across the whole genome contribute to the hazard ratio of transition between different stages of smoking behavior by performing time‐to‐event analyses within a large Finnish twin family cohort (*N* = 1962), and further conducted mediation analyses of plausible intermediate traits for significant SNPs.

**Results:**

Genome‐wide significant signals were detected for three of the four transitions: (1) for smoking cessation on 10p14 (*P* = 4.47e‐08 for rs72779075 flanked by *RP11‐575N15* and *GATA3*), (2) for tolerance on 11p13 (*P* = 1.29e‐08 for rs11031684 in *RP1‐65P5.1*), mediated by smoking quantity, and on 9q34.12 (*P* = 3.81e‐08 for rs2304808 in *FUBP3*), independent of smoking quantity, and (3) for smoking initiation on 19q13.33 (*P* = 3.37e‐08 for rs73050610 flanked by *TRPM4* and *SLC6A16*) in analysis adjusted for first time sensations. Although our top SNPs did not replicate, another SNP in the *TRPM4‐SLC6A16* gene region showed statistically significant association after region‐based multiple testing correction in an independent Australian twin family sample.

**Conclusion:**

Our results suggest that the functional effect of the *TRPM4‐SLC6A16* gene region deserves further investigation, and that complex neurotransmitter networks including dopamine and glutamate may play a critical role in smoking initiation. Moreover, comparison of these results implies that genetic contributions to the complex smoking behavioral phenotypes vary among the transitions.

## Introduction

Various pivotal stages can be identified in an individual's smoking history, including smoking initiation, conversion from experimenting to established use, development of tolerance, and cessation. Each transition is likely influenced by environmental and genetic factors, some of which are common to all steps, and others that are specific. Modest to high heritability has been reported for the majority of smoking behavior phenotypes (Madden et al. 2004; Horimoto et al. 2012; Loukola et al. 2014), with a study of Finnish adult twins reporting heritability estimates of 0.59 in males and 0.36 in females for age at initiation of smoking (Broms et al. 2006).

Nicotine is the main psychoactive compound in tobacco, and exerts its functions by binding to nicotinic acetylcholine receptors (nAChR). Genome‐wide association study (GWAS) meta‐analyses have robustly reported that the *CHRNA5‐CHRNA3‐CHRNB4* nAChR gene cluster on 15q25 and the *CHRNB3‐CHRNA6* region on 8p11.21 are associated with smoking quantity (measured by cigarettes per day, CPD) and nicotine dependence (ND) (measured by the Fagerström Test for Nicotine Dependence, FTND (Heatherton et al. 1991)) (Liu et al. 2010; The Tobacco and Genetics Consortium 2010; Thorgeirsson et al. 2010). However, less than 1% of the variance in the amount smoked is explained by alleles of these genes, with an average effect per allele of one CPD. Age of onset phenotypes have been utilized in some targeted studies of nAChR genes. Variants in the *CHRNA5‐CHRNA3‐CHRNB4* gene cluster are shown to predict a later age of smoking cessation (Chen et al. 2012), and the effect of a functional *CHRNA5* variant (rs16969968) on smoking quantity is reported to be stronger in early‐onset smokers than in late‐onset smokers (Hartz et al. 2012). Further, a genetic risk score composed of *CHRNA5‐CHRNA3‐CHRNB4* and *CYP2A6* (encoding the main metabolic enzyme for nicotine) variants highlighted in large CPD GWAS meta‐analyses (Liu et al. 2010; The Tobacco and Genetics Consortium 2010; Thorgeirsson et al. 2010) was unrelated to smoking initiation, but associated with progression to heavy smoking and ND (Belsky et al. 2013).

Several GWAS have targeted smoking initiation (Vink et al. 2009; The Tobacco and Genetics Consortium 2010; Thorgeirsson et al. 2010; Siedlinski et al. 2011; Argos et al. 2014) or cessation (The Tobacco and Genetics Consortium 2010; Siedlinski et al. 2011; Argos et al. 2014) with phenotypes dichotomized into ever versus never or used as quantitative age of onset phenotypes. Only the large GWAS meta‐analysis of the Tobacco and Genetics Consortium yielded signals in tyrosine kinase and dopamine signaling pathway genes that genome‐wide significantly associated with smoking initiation (never vs. ever smokers) and smoking cessation (former vs. current smokers), respectively (The Tobacco and Genetics Consortium 2010). Time‐to‐event analysis is more powerful than analysis of binary traits or transformed quantitative phenotypes because it incorporates information of follow‐up time span and allows for censoring. There is a huge gap in understanding the contribution of an associating variant to a specific trait. Causal mediation analysis has been used to improve the understanding of the mechanisms underlying detected associations (Jiang et al. 2013; Liu et al. 2013). The estimation of mediation effects in the context of survival models has been discussed in previous literature (Lange and Hansen 2011; VanderWeele 2011; Nemes et al. 2013). Smoking behavior is likely influenced by a variety of additional factors besides the function of nicotinic receptors and nicotine metabolism, such as psychiatric disorders (e.g., schizophrenia, depression) and somatic consequences of smoking (e.g., bronchitis, chronic obstructive pulmonary disease). Understanding the mechanisms underlying the progression of smoking behavior could facilitate the development of targeted cessation pharmacotherapies and interventions.

In this study, we investigated which SNPs across the whole genome contribute to the speed of transition between different stages of smoking behavior by performing time‐to‐event analyses within a large Finnish twin family cohort (*N* = 1962). We tracked and elaborately recorded smoking history by detailed interviews. We adopted time‐to‐event random effects models to examine the rate at which the smokers proceed to the next stage, and incorporated a kinship matrix to account for the family structure. Specifically, we tested whether genetic variants are associated with a younger age at smoking initiation, speed of transition to daily smoking (dichotomized into rapid vs. slow progression), speed of transition from daily smoking to the period of heaviest smoking, and earlier quitting from smoking. When performing association analyses, we considered plausible intermediate traits as covariates. We then investigated whether the independent variable, that is, a SNP, affects the outcome independently or influences the mediators, which in turn affects the outcome.

## Materials and Methods

### Sample

**Figure 1:**
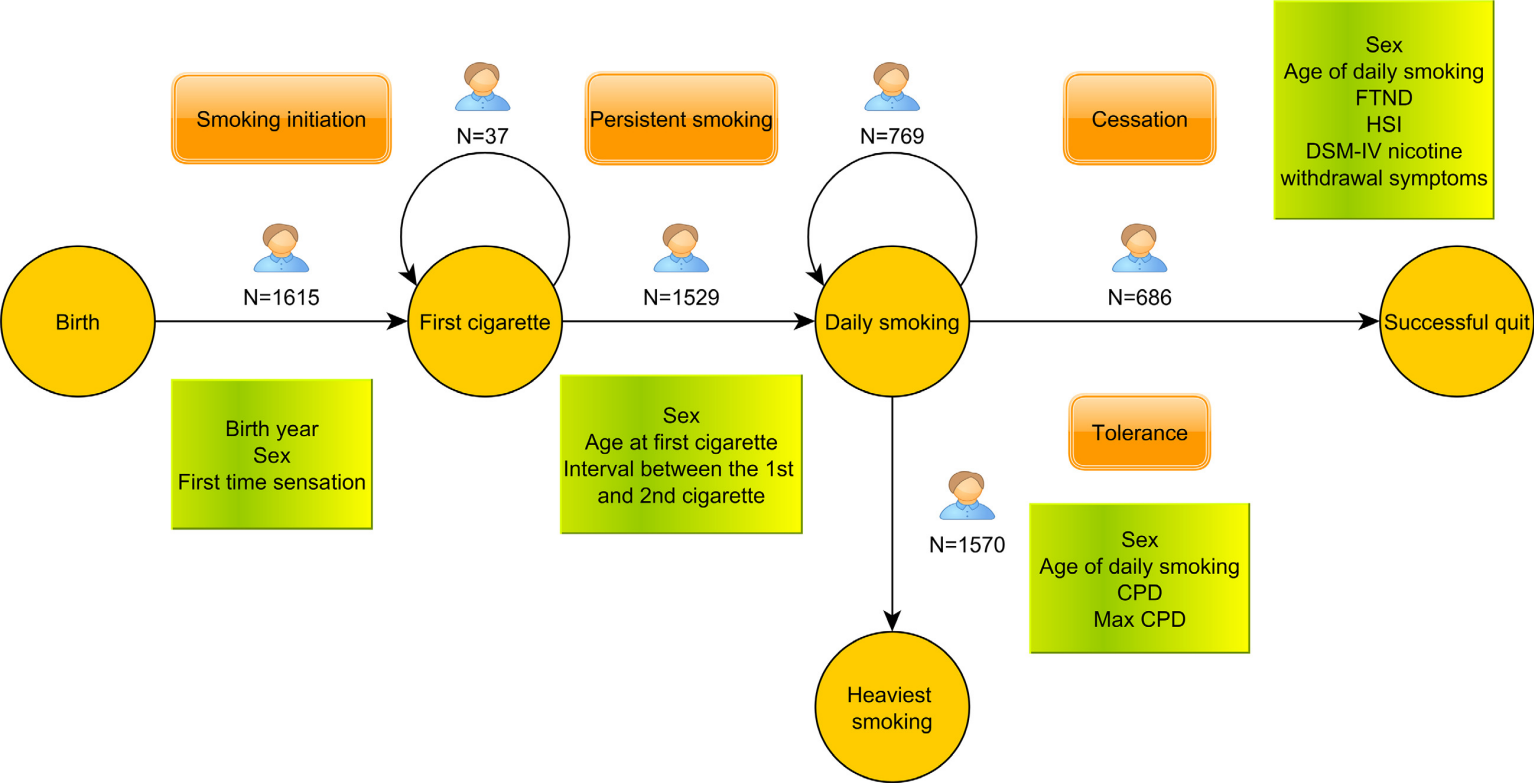
A flowchart of the study design. Circle: smoking stage; orange rectangle: milestone in smoking behavior; green rectangle: considered covariates. Numbers indicate the number of subject included in the analysis with sex as a covariate for each transition (for Smoking initiation also birth year was included).

Data collection has been described in previous publications (Broms et al. 2007; Loukola et al. 2014). Briefly, subjects were ascertained from the Finnish Twin Cohort study encompassing 35,834 twins born in 1938–1957. Twin pairs concordant for smoking were recruited, along with their family members (mostly siblings). Altogether 1962 subjects (mean age 56.2 ± 8.3, 50.9% men) from 734 families were included in this study, consisting of 858 subjects from 429 full dizygotic (DZ) pairs, 146 additional DZ subjects (one co‐twin per pair), 131 monozygotic (MZ) subjects (one co‐twin per pair), 19 twin subjects (unknown zygosity) without co‐twins, 681 siblings (brothers and sisters), and 127 parents (one parent per family). All the subjects had initiated smoking and smoked on average 15 CPD. Altogether 880 subjects were successful quitters defined by self‐reported abstinence of at least 6 months at the time of the interview. Written informed consent was obtained from all subjects who were interviewed and/or gave DNA samples before the beginning of the studies. The collection of the informed consent as well as blood samples followed the recommendations given in the Declaration of Helsinki and its amendments. Data collection was approved by the hospital district of Helsinki and Uusimaa, the ethical committee for epidemiology and public health (HUS 136/E3/01). A flowchart of the overall study design is shown in Figure 1.

### Replication sample

For replication of the detected associations, we utilized an independent Australian twin family sample (NAG‐OZALC, *N* = 1884, *N* = 3389, and *N* = 2723, for initiation, tolerance, and cessation analyses, respectively). A brief sample description is presented in the Table S1; detailed sample description has been previously published elsewhere (Heath et al. 2011).

### Phenotypes

Detailed information on the evolution of smoking behavior as well as relevant covariates was retrospectively collected through structured telephone interviews, as previously described (Loukola et al. 2014). Questions used to inquire the several milestones in smoking behavior are presented in Table 1. We constructed four time‐to‐event phenotypic variables based on the transitions measured in years between progressive smoking states: (1) *smoking initiation* (years from birth to the age of smoking the first cigarette), (2) *persistent smoking* (years from the age of smoking the first cigarette to the age of daily smoking), (3) *tolerance* (years from the age of daily smoking to the age when the heaviest smoking started), and (4) *cessation* (years from the age of daily smoking to the age of successful quitting). For smoking cessation, we defined continuous abstinence of more than 6 months as successful quitting, as previously suggested (Hughes et al. 2003), and those individuals still smoking by the time of the interview were treated as censored. We excluded subjects (*N* = 70) abstinent for less than 6 months prior to the interview because their cessation status cannot be deduced. Further, we excluded 55 subjects who reported quitting due to health reasons, as genetic background may have little or no effect on quitting success in such a situation. The basic characteristics of the data used in the analyses are listed in Table 2.

**Table 1:** Questions used to assess the milestones and potential confounders of smoking behavior

	Questions used	Mean (±SD)
**Smoking state**		
Age of smoking the first cigarette	“How old were you when you smoked a whole cigarette for the first time?”	16.06 (±4.59)
Age when daily smoking started	“How old were you when you first smoked cigarettes daily or almost daily and at least for 2 months?”	18.59 (±5.00)
Age when the heaviest smoking started	“How old were you when the period of heaviest smoking started?”	26.60 (±9.93)
Age of successful quitting	“Do you still smoke or have you quit?”; if one replies “has quit”, then ask “When did you last smoke (even a puff)?”; if one replies “Last puff >6 months ago”, then ask “How old were you when you last smoked (even a puff)”?	46.14 (±12.20)
**Covariate**		
Cigarettes per day (CPD)	“How many cigarettes do you/did you used to smoke per day?” 8 response categories (1–2, 3–5, 6–10, 11–15, 16–19, 20–25, 26–39, and 40 + ), original categorical observations were replaced with class means of CPD (1.5, 3.5, 8, 13, 17.5, 22.5, 32.5, and 45 CPD, respectively)	14.95 (±9.25)
Maximum cigarettes per day (max CPD)	“What is the maximum number of cigarettes you have ever smoked during 1 day (24‐h period)?”	28.77 (±14.41)
Positive first time sensations	Sum score of three questions measuring sensation felt after smoking the first cigarette or first puffs (“While smoking your very first cigarettes, did you (1) like the taste or smell of the cigarette, (2) feel more relaxed, (3) feel a pleasurable rush or buzz?”) (range 1–10)	3.06 (±2.66)
Negative first time sensations	Sum score of seven questions measuring sensation felt after smoking the first cigarette or first puffs (“While smoking your very first cigarettes, did you (1) cough, (2) feel dizzy or light headed, (3) get a headache, (4) feel your heart racing, (5) feel nauseated, (6) feel your muscles tremble or become jittery, (7) feel burning in your throat”?) (range 1–13)	7.06 (±2.61)
Fagerström Test for Nicotine Dependence (FTND) Score	Score based on (Heatherton et al. 1991) (range 0–10)	3.53 (±2.39)
Years between the first and second cigarettes	“After you smoked a whole cigarette for the first time, how long did it take until you smoked another whole cigarette?) nine response categories ((A) never, (B) same day, (C) next day, (D) within a week, (E) within a month, (F) within 3 months, (G) within 6 months, (H) within a year, (I) after over a year); response alternatives were further categorized into three categories: (2)= smoked the second cigarette same or next day, (1)= it took longer than 1–2 days to smoke the second cigarette, (0)= never smoked another cigarette	1.52 (±0.51)
Diagnostic and Statistical Manual of Mental Disorders, 4th Edition (DSM‐IV) nicotinewithdrawal symptoms	The participants were queried about each DSM‐IV nicotine withdrawal symptom: irritability, restlessness, concentration problems, depressed mood, increased appetite, sleep problems, nervousness, and decreased heart rate (Association 2000), within the context of a smoking cessation. The reported symptoms were summed up to form a symptom score (range 0–8)	2.32 (±2.08)

**Table 2:** Basic characteristics of the datasets from the Finnish Twin cohort used in the four smoking behavior transition analyses

	Start of follow‐up	End of follow‐up	Mean age at the end of follow‐up	Mean duration in years^b^	Sample size	Number of families	Percentage of males	Covariates^a^
Smoking initiation	Birth	Age at the first cigarette	16.05 ± 4.59	16.05 ± 4.59	1615	701	56.6%	Sex (*P* < 1e‐10), birth year (*P* < 1e‐10)
			16.05 ± 4.60	16.05 ± 4.60	1530	688	56.9%	Sex (*P* < 1e‐10), positive first time sensations (*P* = 1.1e‐03), negative first time sensations (*P* = 3.5e‐07), birth year (*P* < 1e‐10)
Tolerance	Age of daily smoking	Age when heaviest smoking started	26.59 ± 9.92	8.10 ± 9.57	1570	719	57.4%	Sex (*P* = 0.77)
			26.59 ± 9.92	8.10 ± 9.57	1570	719	57.4%	Sex (*P* = 2.1e‐08), CPD (*P* = 4.4e‐04), max CPD (*P* = 1.2e‐09), age of daily smoking (*P* = 2.1e‐08)
Cessation	Age of daily smoking	Age of quitting or being censored at the date of interview	38.85 ± 11.45	20.23 ± 11.19	1455	708	56.8%	Sex (*P* = 0.014)
			38.64 ± 11.39	20.29 ± 11.15	1431	704	57.2%	Sex (*P* = 1.2e‐05), FTND (*P* < 1e‐10), DSM‐IV nicotine withdrawal symptoms (*P* = 9.0e‐03)

Binary outcome analysis using generalized linear mixed‐effects model:							
	Outcome			Sample size	Number of families	Percentage of males	Covariates^a^
Persistent smoking	Daily smoking started within 1 year (yes or no) after the first cigarette^c^			1529^c^	711	57.6%	Sex (*P* = 3.22e‐05)
				1522	710	57.4%	Sex (*P* = 0.025), age at the first cigarette (*P* = 8.7e‐15), interval between the first and second cigarette (*P* < 1e‐10)

CPD, cigarettes per day; FTND, Fagerström Test for Nicotine Dependence; DSM‐IV, Diagnostic and Statistical Manual of Mental Disorders, 4th Edition.

The sample sizes for each transition analysis differ slightly because any subject with missing data on the corresponding phenotype or covariates was excluded from the analysis of that transition.

a *P*‐values obtained from the coxme package or GLMM.

b Censored individuals are not included in the computation of mean age at the end of follow‐up and mean duration in year.

c Out of the 1529 subjects, 450 proceeded to daily smoking within one year, while for 1079 subjects it took over a year. In our data set additional 37 subjects did not proceed to daily smoking during the follow‐up period.

### Evaluation of Covariates

We first performed genome‐wide time‐to‐event analyses for each of the transitions with only sex as a covariate. As an exception, for smoking initiation we also included another genotype‐independent covariate, birth year, as the age of smoking initiation of an individual may be affected by the specific environment in his/her generation (Morabia et al. 2002). As a follow‐up analysis, we then conducted another set of genome‐wide time‐to‐event analyses with additional transition‐specific intermediate covariates in order to obtain comparable results for the following mediation analysis of significant SNPs, and to increase the chance of detecting SNPs associated with the transitions independently of the intermediate covariates. We evaluated biologically plausible covariates for each transition (selected based on literature and a priori knowledge of factors affecting smoking behavior), and included those significantly associated with the transition (*P* < 0.05) (Table 2), as only significantly associated covariates are eligible as candidate mediators.

For smoking initiation, we considered positive and negative first time sensations as these variables attempt to capture the individual responses to the first‐ever dose of nicotine, and likely have a significant effect on the probability of smoking a whole cigarette (Rios‐Bedoya et al. 2009).

For persistent smoking, we considered age at the first cigarette, as it significantly affects the downstream steps in smoking behavior (Breslau et al. 1993). Further, the interval between the first and second cigarette was considered as an estimate of the initial speed of transition. However, as the genome‐wide analyses showed lack of power, no follow‐up analysis was performed.

For tolerance, we considered age of initiation of daily smoking, as it is shown to predict earlier age at heaviest smoking (Kendler et al. 2013). Further, we considered CPD, max CPD ever smoked during a 24 h period, and CPD at the period of heaviest smoking, as smoking quantity may affect the development of tolerance; rodent studies show that rapid tolerance is related to frequency of nicotine administration and dose (Aceto et al. 1986).

For smoking cessation, we considered FTND and DSM‐IV nicotine withdrawal symptoms, as they likely affect the ability to quit (Kozlowski et al. 1994).

### Genotyping and quality control

Genotyping was performed at the Wellcome Trust Sanger Institute using the Human670‐QuadCustom Illumina BeadChip (*N* = 1104) and the Illumina Human Core Exome BeadChip (*N* = 858). Pre‐imputation exclusion criteria for the data generated with the Human670‐QuadCustom Illumina BeadChip were minor allele frequency (MAF) < 0.01, sample and SNP call rate <0.95 (<0.99 for SNPs with MAF < 0.05); and the criteria for the data generated with the Illumina Human Core Exome BeadChip were minor allele count <2, sample call rate <0.98, SNP call rate <0.95 (<0.99 for SNPs with MAF < 0.05). Both genotype datasets were filtered according to the Hardy–Weinberg equilibrium (HWE) test *P* < 1e‐06. Further, sample heterozygosity test, gender, and Multidimensional Scaling (MDS) outlier checks were done for both. Pre‐phasing of the data was done with SHAPEIT2 (Delaneau et al. 2013) and imputation with IMPUTE2 (Howie et al. 2009) using the 1000 Genomes Phase I integrated haplotypes (produced using SHAPEIT2) reference panel (1000 Genomes Project Consortium, 2012). Quality controls and imputation for the GWAS data were done centrally at the Institute for Molecular Medicine Finland (FIMM), University of Helsinki, Helsinki, Finland.

### Statistical analyses

The genome‐wide time‐to‐event analyses for smoking initiation, tolerance, and cessation were performed using the Cox proportional hazards (PH) model (Cox 1972) with random effects. We calculated the empirical kinship matrix based on the observed relationship of family members, and employed the coxme R package (Therneau 2012) which implements the Cox PH model with random effects of multivariate normal distribution by utilizing penalized partial likelihood (Ripatti and Palmgren 2000). The selected sample with only one co‐twin from each MZ pair and at most one parent in each family resulted in a kinship matrix in which individuals in a family share the same genetic correlation coefficient, dramatically reducing the computational time of the coxme function. For persistent smoking, we found that over a quarter of individuals (*N* = 450) became daily smokers within 1 year, and those who did not engage in daily smoking within 20 years since initiation were considered as long‐term survivors. In order to account for this, we first adopted and implemented a mixture cure model to analyze this transition in the context of survival framework (Yu and Peng 2008). The results, however, showed an inflated false positive error rate (data not shown) probably due to the potential inaccuracy of the variance estimation as shown in previous simulation studies (Yu and Peng 2008). We therefore addressed this issue by dichotomizing the survival time variable and classified those becoming a daily smoker within 1 year after smoking the first cigarette as rapid progression (*N* = 450), and those becoming a daily smoker after > 1 year as slow progression (*N* = 1079). We then conducted the association analysis on this binary variable with the logistic linear mixed effects model implemented in the glmmML R package (Broström and Holmberg 2011).

We performed the single‐variant association analyses only for those SNPs with a MAF > 5% and HWE test *P* > 1e‐05, and ensured that identified top signals had high imputation information score (>0.8). The total number of SNPs included in the genome‐wide time‐to‐event analyses was 5,918,992. We checked for potential population stratification by investigating the principal components for the family founders with the EIGENSOFT package (Price et al. 2006); no outliers with foreign ancestry were found, as was expected as the twin data consist purely of native Finnish population. We adopted the genome‐wide significance *P*‐value of *P* < 5e‐08 as a cutoff, which has been broadly recognized as a criterion based on the sequencing data of European populations (Sham and Purcell 2014). For chromosomes containing loci exceeding the cutoff, we performed conditional analyses where we adjusted for the top SNP to test whether the detected association represented an independent signal. We list top five SNPs, regardless of their *P*‐values, to allow for comparison with results from the follow‐up studies adjusting for the relevant covariates. For SNPs identified as significantly associated with the transitions, we further performed mediation analyses to investigate whether the association is through plausible mediators. Details of the mediation analyses are presented in the Appendix S1. We used GWAVA (Ritchie et al. 2014) for predicting the potential functional effects of the associating noncoding region SNPs.

For genome‐wide significant signals, replication was attempted for the top five SNPs as well as with all SNPs within the nominated genes (with ±50 kb flanking regions). For the replication analyses, models identical to those applied in the discovery sample were used. To account for multiple testing, we applied a modified Bonferroni correction based on the effective number of independent SNPs in the gene regions calculated using a formula proposed by Gao and colleagues (Gao et al. 2008; Hendricks et al. 2014).

We further conducted fixed‐effect meta‐analyses for the top five SNPs based on the effect sizes and standard errors from the discovery and replication studies using GWAMA (http://www.well.ox.ac.uk/gwama). *P*‐values below 5e‐08 were considered statistically significant.

## Results

### Genome‐wide time‐to‐event analysis of smoking initiation

The top five SNPs for the age at the first cigarette are listed in Table 3. In the time‐to‐event analysis rs73050610 on 19q13.33 was highlighted (*P* = 1.12e‐07) (Fig. S1, Table 3). In a follow‐up analysis additionally adjusted for first time sensations rs73050610 achieved genome‐wide significance (*P* = 3.37e‐08) (Fig. S2, Table 3). The LD block in which all top five SNPs are located is flanked by genes *TRPM4* (1 kb apart) and *SLC6A16* (60 kb apart). A regional plot of the 19q13.33 locus is shown in Figure S3. In an analysis conditioned on rs73050610, no residual genome‐wide significant signal remained, suggesting that there is only one independent signal in this locus. The hazard ratio (HR) of rs73050610 is 0.80, suggesting that carriers of the minor allele have a 20% lower hazard per allele of smoking the first whole cigarette.

**Table 3:** The top five SNPs from the genome‐wide time‐to‐event analyses

CHR	BP	*P*	HR (or OR)	SNP ID	A1	A2	AF (CEU)	HWE	Info	*P* ^r^	HR^r^	*P* ^m^
Smoking initiation with sex and birth year												
19	49721561	1.116e‐07	0.800	rs73050610	T	C	0.485 (0.441)	0.669	0.983	0.899	1.005	4.170e‐04
17	64169182	1.296e‐07	1.676	rs75395715	C	T	0.056 (0.041)	0.683	0.863	0.602	0.942	2.380e‐04
19	49728893	2.475e‐07	0.806	rs8105169	G	C	0.488 (0.435)	0.552	0.998	0.910	1.004	1.187e‐03
19	49727160	2.486e‐07	0.806	rs8112298	T	C	0.488 (0.435)	0.562	0.999	0.918	1.004	6.020e‐04
19	49725042	2.531e‐07	0.806	rs8103217	A	G	0.488 (0.435)	0.572	0.999	0.921	1.004	4.630e‐04
Smoking initiation with sex, birth year, positive and negative first time sensations												
19	49721561	**3.374e‐08**	0.783	rs73050610	T	C	0.485 (0.441)	0.669	0.983	0.771	0.988	4.920e‐05
19	49725042	6.256e‐08	0.788	rs8103217	A	G	0.488 (0.435)	0.572	0.999	0.756	0.987	8.400e‐05
19	49727160	6.289e‐08	0.788	rs8112298	T	C	0.488 (0.435)	0.562	0.999	0.756	0.987	9.470e‐05
19	49728893	6.307e‐08	0.788	rs8105169	G	C	0.488 (0.435)	0.552	0.998	0.763	0.988	9.590e‐05
19	49728186	6.604e‐08	0.788	rs3843746	C	T	0.489 (0.441)	0.587	0.997	0.757	0.987	9.650e‐05
Persistent smoking with sex												
1	214682808	6.683e‐06	1.837	rs6701211	G	A	0.088 (0.076)	0.515	0.997	NA^a^	NA^a^	NA
17	1697361	6.909e‐06	1.554	rs12941003	G	A	0.302 (0.429)	0.641	0.800	NA^a^	NA^a^	NA
10	128564711	6.971e‐06	1.562	rs4962638	G	A	0.750 (0.588)	0.607	0.994	NA^a^	NA^a^	NA
14	96402039	8.570e‐06	0.625	rs941777	A	T	0.828 (0.718)	0.333	0.995	NA^a^	NA^a^	NA
14	96402810	9.585e‐06	0.627	rs7155176	C	T	0.827 (0.718)	0.407	0.996	NA^a^	NA^a^	NA
Persistent smoking with sex, age at the first cigarette, years between the first and second cigarettes												
14	96402039	7.074e‐06	0.592	rs941777	A	T	0.828 (0.718)	0.333	0.995	NA^a^	NA^a^	NA
14	96404277	7.118e‐06	0.594	rs1957126	T	C	0.827 (0.706)	0.467	0.995	NA^a^	NA^a^	NA
14	96403278	7.580e‐06	0.595	rs6575554	T	G	0.827 (0.706)	0.489	0.996	NA^a^	NA^a^	NA
14	96402810	8.260e‐06	0.595	rs7155176	C	T	0.827 (0.718)	0.407	0.996	NA^a^	NA^a^	NA
14	96402094	8.691e‐06	0.596	rs1957127	G	A	0.827 (0.718)	0.407	0.997	NA^a^	NA^a^	NA
Tolerance with sex												
11	32293139	**1.294e‐08**	1.460	rs11031684	T	G	0.124 (0.153)	0.981	0.838	NA^b^	NA^b^	NA
9	133510021	5.499e‐07	1.251	rs2304808	T	C	0.251 (0.218)	0.343	0.984	0.161	1.051	2.570e‐05
6	91995475	5.825e‐07	1.293	rs1884258	G	A	0.160 (0.076)	0.521	0.996	0.404	0.958	3.262e‐03
22	23058620	8.340e‐07	1.386	rs9620160	A	G	0.129 (0.135)	0.567	0.919	NA^b^	NA^b^	NA
9	133488447	1.481e‐06	1.230	rs2304812	G	A	0.272 (0.235)	0.550	1.000	0.266	1.038	1.260e‐04
Tolerance with sex, age of daily smoking, CPD, and max CPD												
9	133510021	**3.811e‐08**	1.307	rs2304808	T	C	0.251 (0.218)	0.343	0.984	0.108	1.066	2.350e‐06
9	133490496	1.037e‐07	1.306	rs7040341	G	T	0.237 (0.200)	0.164	1.000	0.216	1.054	1.190e‐05
9	133492544	1.318e‐07	1.281	rs28476634	A	T	0.272 (0.235)	0.550	0.999	0.208	1.049	1.740e‐05
9	133488447	1.321e‐07	1.281	rs2304812	G	A	0.272 (0.235)	0.550	1.000	0.213	1.049	1.690e‐05
9	133489452	1.424e‐07	1.281	rs11795269	C	T	0.272 (0.235)	0.614	0.999	0.191	1.051	1.550e‐05
Cessation with sex												
10	8841891	**4.473e‐08**	1.479	rs72779075	C	A	0.207 (0.088)	0.587	0.994	0.380	1.084	1.220e‐06
10	8787478	1.028e‐07	0.683	rs11255894	G	T	0.795 (0.731)	0.657	0.941	0.214	0.910	2.360e‐06
10	8838696	1.351e‐07	1.457	rs7072531	C	T	0.210 (0.094)	0.386	0.989	0.236	1.099	2.440e‐06
10	8838279	1.374e‐07	1.457	rs112340507	T	G	0.210 (0.094)	0.403	0.989	0.245	1.096	2.840e‐06
10	8791773	1.505e‐07	0.688	rs1413687	A	T	0.792 (0.912)	0.336	0.991	NA^b^	NA^b^	NA
Cessation with sex, DSM‐IV nicotine withdrawal, FTND												
10	8841891	1.829e‐07	1.447	rs72779075	C	A	0.207 (0.088)	0.587	0.994	0.328	1.098	1.850e‐06
10	8838696	7.661e‐07	1.419	rs7072531	C	T	0.210 (0.094)	0.386	0.989	0.210	1.109	4.980e‐06
10	8838279	7.850e‐07	1.419	rs112340507	T	G	0.210 (0.094)	0.403	0.989	0.226	1.104	6.130e‐06
1	157267069	8.272e‐07	0.689	rs6427366	T	A	0.781 (0.835)	0.682	0.906	0.429	0.941	4.760e‐05
10	8791773	9.169e‐07	0.706	rs1413687	A	T	0.792 (0.912)	0.336	0.991	NA^b^	NA^b^	NA

CHR, chromosome; BP, base pair position according to build 37; *P*,* P*‐value (*P*‐values exceeding the genome‐wide significance threshold [*P* < 5e‐08] are highlighted in bold); SNP ID, rs‐number; A1, non‐effect allele; A2, effect allele; AF, allele frequency of A2 observed in this study; CEU, allele frequency of A2 from the 1000 Genomes Project phase I of Utah Residents (CEPH) with Northern and Western European ancestry; HWE, Hardy–Weinberg Equilibrium test *P*‐value; Info, measure of the observed statistical information associated with the allele frequency estimate (i.e., imputation info score); *P* ^r^, *P*‐value from the Australian replication study (rs11031684 failed imputation QC. Rs9620160 and rs1413687 were not available in the Australian 1000 Genomes imputed dataset); HR^r^, HR from the Australian replication study; *P* ^m^, *P*‐value from the meta‐analyses; CPD, cigarettes per day; FTND, Fagerström Test for Nicotine Dependence; DSM‐IV, Diagnostic and Statistical Manual of Mental Disorders, 4th Edition.

a Replication was not attempted as the association in the discovery sample was not genome‐wide significant.

b SNP not available in the replication sample.

None of the 19q13.33 top five SNPs showed statistically significant evidence for replication in an independent Australian twin family sample; however, the effect sizes shared the same direction in analyses adjusted for first time sensations. When attempting replication with all SNPs located in *TRPM4* and *SLC6A16* (with ±50 kb flanking regions), statistically significant association was seen in analyses adjusted for first time sensations for rs352813 (*P* = 9.2e‐04, surpassing the significance threshold of *P* = 9.43e‐04 based on the modified Bonferroni correction), located 30 kb from the top SNP rs73050610 (Table S2). Rs352813 was not statistically significant in the Finnish sample, and the direction of effect of rs352813 was different in the Australian sample when compared to the Finnish sample. Altogether 18 SNPs in 19q13.33 showed some association (*P* < 0.05) in both populations. Meta‐analysis of the top five SNPs did not yield genome‐wide statistically significant signals.

The estimated average causal mediation effects (ACME) of rs73050610 through the positive and negative first time sensations were 0.0213 (*P* = 0.70) (a positive coefficient from the mediation analysis means that the hazard is decreased. Refer to the Appendix S1 for the details of the mediation analyses) and −0.0291 (*P* = 0.77), respectively, suggesting that the effect of rs73050610 is not mediated through the positive or negative first time sensations.

### Genome‐wide time‐to‐event analysis of persistent smoking

The top five SNPs for the transition from the age at the first cigarette to the age of daily smoking (rapid vs. slow transition) are shown in Table 3. None of the SNPs exceeded or approached the genome‐wide significance threshold. Manhattan and Q–Q plots are presented in Figure S4. The Q–Q plots show deflated *P*‐values indicating lack of sufficient statistical power, likely due to the limited sample sizes for an association analysis with a binary variable. We did not pursue follow‐up analyses or attempt replication for this transition.

### Genome‐wide time‐to‐event analysis of tolerance

The top five SNPs for the transition from daily smoking to heaviest smoking are presented in Table 3. In the time‐to‐event analysis rs11031684 on 11p13 showed genome‐wide significant association (*P* = 1.29e‐08) (Fig. S5, Table 3) with an HR of 1.46, suggesting that the minor allele accelerates the progression to tolerance. This SNP is located in a pseudogene *RP1‐65P5.1*, and is flanked by *RCN1* and *WT1* within a distance of approximately 150 kb. A regional plot of the 11p13 locus is shown in Figure S6. In an analysis conditioned on rs11031684, no residual genome‐wide significant signal remained, suggesting that there is only one independent signal in this locus. In a follow‐up analysis additionally adjusted for age of daily smoking, CPD, and max CPD, rs2304808 residing in *FUBP3* on 9q34.12 showed genome‐wide significant association (*P* = 3.81e‐08) (Fig. S7, Table 3) with an HR of 1.31, suggesting that carriers of the minor allele progress more quickly to tolerance. A regional plot of the 9q34.12 locus is shown in Figure S8. In an analysis conditioned on rs2304808, no residual genome‐wide significant signal remained, suggesting that there is only one independent signal in this locus. None of the top five SNPs replicated in an independent Australian twin family sample, nor did any SNPs located within *RP1‐65P5.1*,* WT1*,* RCN1*, or *FUBP3*. Meta‐analysis of the top five SNPs did not yield genome‐wide statistically significant signals.

The estimated ACME of rs11031684 through CPD and max CPD were −0.525 (*P* = 0.04) and −0.619 (*P* = 0.07), respectively, suggesting that some of the effects of rs11031684 is mediated through CPD. Additionally, we found that rs11031684 was nominally associated with CPD (*P* = 0.0084) and max CPD (*P* = 0.0062). The estimated ACME of rs2304808 through CPD and max CPD were 0.231 (*P* = 0.22) and 0.304 (*P* = 0.24), respectively, suggesting that the effect of rs2304808 is not mediated through CPD or max CPD.

### Genome‐wide time‐to‐event analysis of cessation

The top five SNPs for the transition between daily smoking and cessation are presented in Table 3. In the time‐to‐event analysis rs72779075 on 10p14 showed genome‐wide significant association (*P* = 4.47e‐08) (Fig. S9, Table 3) with an HR of 1.48, suggesting that carriers of the minor allele quit earlier than noncarriers. In a follow‐up analysis additionally adjusted for FTND and DSM‐IV nicotine withdrawal symptom score, rs72779075 remained as the top SNP but the signal no longer was significant (Fig. S10, Table 3). This locus is close to a pseudogene *RP11‐575N15* (with a distance of <40 kb), and the nearest gene is *GATA3* (725 kb apart). A regional plot of the 10p14 locus is shown in Figure S11. In an analysis conditioned on rs72779075, no residual genome‐wide significant signal remained, suggesting that there is only one independent signal in this locus. None of the top five SNPs replicated in an independent Australian twin family sample, nor did any SNPs located within *GATA3*. Meta‐analysis of the top five SNPs did not yield genome‐wide statistically significant signals.

The estimated ACME of rs72779075 through FTND and DSM‐IV nicotine withdrawal symptoms were −4.1744 (*P* = 0.15), and −0.25967 (*P* = 0.55), respectively, suggesting that the effect of rs72779075 is not mediated through ND or nicotine withdrawal symptoms.

### Investigation of previously highlighted smoking‐related genes

To scrutinize whether previously identified smoking‐related genes affect the transitions in the Finnish sample, we investigated common variants (MAF > 5%) surpassing our quality control thresholds from seven relevant gene regions (*CHRNA5‐CHRNA3‐CHRNB4* gene cluster, *CYP2A6, DRD2, DRD4, DBH, CHRNA4,* and *BDNF*). Multiple SNPs in each gene region were nominally associated (*P* < 0.05) with at least one of the transitions (Table S3) although some well‐known SNPs, such as rs16969968 in *CHRNA5*, showed no association (*P* > 0.05) with the transitions, although suggestive association was detected with CPD (*P* = 0.012) and FTND (*P* = 0.0001). Furthermore, SNPs in *DBH* were involved in all four transitions, and rs6011794 in *CHRNA4* was associated with initiation (*P* = 0.0469), tolerance (*P* = 0.0217), and cessation (*P* = 0.0331). Rs2086484 in *CHRNB4*, rs1611121 in *DBH*, and seven SNPs (rs144298540, rs62206942, rs117589312, rs59073906, rs58253278, rs112265183, and rs116920489) in *CHRNA4* showed evidence of association with two transitions. Two SNPs (rs7260629 in *CYP2A6* and rs75298795 in *BDNF*) associated with initiation (*P* = 2.45e‐03) and tolerance (*P* = 2.42e‐03), respectively, with *P*‐values surpassing the significance thresholds (*P* = 2.94e‐03 and *P* = 2.78e‐03, respectively) based on the modified Bonferroni correction.

## Discussion

The progression of smoking behavior from initiation to persistent smoking or cessation is a complex process involving multiple factors. Although some of the genetic factors related to smoking quantity and ND have been identified, we have only began to understand the underlying genome‐wide genetic effects on the development of smoking behavior. In this study, we identified novel SNPs associated with three specific transitions in smoking behavior in a Finnish twin family sample (*N* = 1962).

Considering age at smoking initiation we found that Finnish Twin Cohort subjects born at later decades began smoking at younger age compared to subjects born at earlier decades, which is consistent with a previous Swiss study (Morabia et al. 2002), and that females started smoking later than males, which is also in accordance with previous findings (Okoli et al. 2013). This is also consistent with the evolution of tobacco use in Finland in the 20th century. Although the Q–Q plots (Figs S1 and S2) suggest that the family correlation structure was well controlled for, a mild overdispersion is observed. This implies that common environmental factors may also play a role in smoking initiation even after adjusting for birth year. DZ twins share more environmental factors than non‐twin members of a family, and this environmental correlation structure is not captured by the used kinship matrix, which may lead to slight *P*‐value inflation.

In the time‐to‐event analysis of age at smoking initiation adjusted for first time sensations, multiple SNPs on 19q13.33*,* flanked by *TRPM4* and *SLC6A16*, were highlighted in both the Finnish sample and the independent Australian sample. The highlighted SNPs from the two studies differed but intertwined with each other, providing motivation for further investigating the involvement of nearby genes in smoking initiation. The associating SNPs seem to have a heterogeneous effect. The top five SNPs from the Finnish sample showed no statistically significant association in the Australian sample although they shared the same direction of effect. On the other hand, in the replication sample, a SNP on 19q13.33, located 30 kb from the top SNP in the Finnish sample, showed association adjusted for multiple testing, with a direction of effect opposite from that seen in the Finnish sample. The heterogeneous effects of these markers may reflect variation in LD block structures (Rosenberg et al. 2010) or be due to gene‐environment interactions which substantially amplify the difference of SNP effects, and may suggest that these population‐specific interactions play a critical role in the complex behavioral phenotypes, as previously suggested (Adeyemo and Rotimi 2010; Ho et al. 2010).

First time sensations plausibly affect the probability of smoking a whole cigarette; in line with this, in our study sample positive and negative first time sensations are associated with earlier and later age of initiation, respectively. In order to evaluate whether the association on 19q13.33 was independent of first time sensations, we included them as covariates in the follow‐up analysis, and detected genome‐wide significant association. Further, mediation analysis confirmed that the effect of the top SNP, rs73050610, is independent of first time sensations, and thus the effect is likely due to other mechanisms besides the initial sensations experienced after the first‐ever dose of nicotine. The functional annotation with GWAVA suggests that rs3843746, which is in complete LD with rs73050610 in the Finnish population (*D*′ = 1.00, *r* ^2 ^= 0.979), is a CTCF‐binding region variant. CTCF is involved in multiple regulatory influences on expression of genes, suggesting that the highlighted SNP may have a role in regulating nearby genes. Both of the flanking genes have functions relevant for smoking behavior. *TRPM4* is a calcium‐activated ion channel involved in many activities including immune response (Guinamard et al. 2010), and is the key gene encoding the channel for most calcium‐activated nonselective cationic currents (*I*_can_) observed in native tissues (Mrejeru et al. 2011). *I*_can_ are involved in the generation of tonic and bursting activity in dopamine neurons (Mrejeru et al. 2011), and the burst firing is proposed to encode a “reward” signal during habit learning and pathological addictions (Phillips et al. 2003). Thus, variants in *TRPM4* may affect smoking behavior through the dopaminergic system.

The other flanking gene, *SLC6A16,* is a member of the Na^+^/Cl^−^ dependent neurotransmitter transporter gene family (Farmer et al. 2000), and little is known about its substrates and functional significance. Another member of the solute carrier gene group, *SLC17A7,* located 211 kb from rs73050610, is a vesicular glutamate transporter previously found to be induced by smoking (Flatscher‐Bader et al. 2008). Blockade of glutamatergic transmission inhibits the rewarding‐enhancing effects of nicotine, thus reducing nicotine‐seeking behavior (Li et al. 2014). Alterations in glutamatergic neurotransmission are involved in several psychiatric disorders, such as schizophrenia and alcohol dependence (Shigeri et al. 2004; Comasco et al. 2014), which also influence smoking behavior; however, in this study we did not assess comorbid psychiatric disorders. Inclusion of such phenotypes in future studies would allow scrutiny of potential pleiotropic effects.

Our analyses of persistent smoking indicate that being a female, smoking the first cigarette at a later age, and having a longer interval between the first and the second cigarette predicts later onset of daily smoking. We detected no genome‐wide significant associations for the transition from the age of smoking the first cigarette to the age when daily smoking started. We likely had insufficient power in the analysis of the binary variable (rapid vs. slow transition) especially when using the mixed effects model. Larger samples are needed to investigate the transition to daily smoking.

Our analyses of tolerance indicate that being a woman and smoking less are related to slower progression to tolerance, while later age of daily smoking predicts acceleration. In the time‐to‐event analysis, we detected a genome‐wide significant association with rs11031684 residing on 11p13 in a pseudogene *RP1‐65P5.1*, and located 100 kb downstream of *WT1*. *WT1* is a transcription factor involved in the regulation of human cell growth and differentiation, and is an established tumor suppressor gene. Exposure to heavy smoking influences the methylation pattern of CpG islands in *WT1* (Bruno et al. 2012), providing a plausible mechanism for smoking induced cancers. In addition to affecting methylation, heavy smoking is suggested to affect the development of tolerance (Aceto et al. 1986). In order to evaluate whether the detected association was independent of smoking quantity, we included measures of smoking quantity as covariates in the follow‐up analysis. The signal on 11p13 no longer was significant at a genome‐wide level (*P* = 4.04e‐06); further, mediation analysis suggested that rs11031684 affects the hazard of progression to tolerance partly through smoking quantity. Interestingly, in the follow‐up analysis a novel genome‐wide significant signal emerged for rs2304808 on 9q34.12 within *FUBP3,* which belongs to a family of homologous gene‐regulatory proteins that regulate many common target genes. Mediation analysis further confirmed that the effect of rs2304808 was not mediated through smoking quantity. Inclusion of smoking quantity as a covariate substantially increased the significance of rs2304808, suggesting that including intermediate covariates may increase the power to detect SNPs that are independent of the covariates and directly associated with the phenotype.

In the analysis of smoking cessation we found that being a female, scoring higher in ND, and stronger nicotine withdrawal symptoms predicts slower transition to quitting, whereas later age of daily smoking predicts faster quitting. In the time‐to‐event analysis we detected genome‐wide significant association with rs72779075 on 10p14, located 35 kb from a pseudogene *RP11‐575N15* and around 725 kb downstream of *GATA3*. In the follow‐up analysis the signal of this SNP was no longer significant; however, our mediation analysis showed no statistically significant evidence for the effect of rs72779075 being mediated by ND or nicotine withdrawal symptoms, and thus the effect on difficulty in achieving and maintaining abstinence is likely due to other mechanisms besides the severity of ND and withdrawal. Interestingly, nicotine upregulates expression of *GATA3* through stimulation of nAChRs (Arredondo et al. 2006). *GATA3* is crucial in inducing allergic airway inflammation (Barnes 2008); although rs72779075 is located 725 kb downstream of *GATA3*, it may tag variants that influence symptoms of airway inflammation and thus may motivate smokers to quit. Alternatively, *RP11‐575N15* may have an unidentified function. Transcripts produced from pseudogenes may, for example, regulate the effects of microRNAs on their targets by competing for microRNA binding (Swami 2010).

Although our data included an extraordinarily detailed smoking history, there are still some limitations in our study. Smoking behavior encompasses psychiatric and social behaviors in which both complex genetic and environmental factors are involved; these were not accounted for in our analyses. Also other plausibly relevant covariates, such as socio‐economic status, and working environment, were not considered. Further, our phenotype data were collected retrospectively in subjects with a mean age of 56 years at the time of the interview; thus the accuracy of self‐reported ages of onsets may be influenced by recall bias. Although the interviews contained detailed measures of ND, the age of onset of ND was not assessed, and thus we were not able to perform time‐to‐event analyses of ND. However, we analyzed tolerance, which is the key dimension of ND. The efficient long‐term survival model accounting for family structure, which to the best of our knowledge is not available, would be more appropriate when analyzing the transitions to daily smoking and quitting, and it should be considered in the future. Future studies should also attempt to investigate low‐frequency variants (MAF < 0.05) in the context of transitions in smoking behavior.

The lack of replication may implicate false positive findings. Alternatively, it may be due to lack of power in the replication sample, population specificity of the associations, as well as gene‐environment interactions. In addition, our study sample comes from one of the best‐characterized founder populations, the Finns. Unique LD patterns are observed in founder populations (Service et al. 2006); thus, the lack of replication may at least partly be due to the genetic heterogeneity between the discovery sample (Finns) and replication sample (Australians). It has been shown that population isolates, especially those founded recently, such as Finland, have longer stretches of LD than outbred populations and may thus achieve better genome‐wide coverage with equivalent numbers of markers (Peltonen et al. 2000; Service et al. 2006). Our top SNPs may tag underlying functional variants in the Finnish sample, but due to differences in LD structures the functional variants are not necessarily captured by these SNPs in the Australian data. Population‐specific functional variants are known to exist (Lim et al. 2014), and one has already been documented in the Finnish population for a behavioral trait (Bevilacqua et al. 2010).

The advantages of this study include the detailed phenotype profiles, allowing us to more precisely handle the complexity of the smoking behavior phenotype, which has previously been modeled in a relatively static way (e.g., ever vs. never smoker). This approach to phenotype refinement may help to identify novel signals, and perhaps be tractable with smaller samples than conventionally required. Our novel results suggest that the various stages in smoking history are affected by different underpinning mechanisms. Complex neurotransmitter networks including dopamine and glutamate may play a critical role in initiation, while airway inflammation possibly contributes to smoking cessation.

In conclusion, we detected genome‐wide significant association between SNPs and three transitions in smoking behavior in a Finnish twin family sample. The interpretation of our findings should be cautious before robust evidence of replication is obtained. Our results are valuable for guiding follow‐up functional analyses, provide valuable clues into the etiology of smoking behavior, and encourage further studies utilizing time‐to‐event phenotypes in addictive behavior.

## Conflict of Interest

The authors declare that they have no competing interests. Kaprio and Korhonen have provided consultation to Pfizer on nicotine dependence and its treatment.

## Supporting information


**Figure S1.** (A) Manhattan and (B) Q–Q plots for the genome‐wide time‐to‐event analysis of smoking initiation (adjusted for sex and birth year) (*λ *= 1.083).

**Figure S2.** (A) Manhattan and (B) Q–Q plots for the follow‐up analysis of smoking initiation (adjusted for sex and birth year, as well as positive and negative sensation scores) (*λ *= 1.089).

**Figure S3.** Regional plot of the 19q13.33 locus rs73050610 identified for smoking initiation (data from analysis adjusted for sex and birth year, as well as positive and negative sensation scores).

**Figure S4.** (A) Manhattan and (B) Q–‐Q plots for the genome‐wide time‐to‐event analysis of persistent smoking (adjusted for sex) (*λ *= 1.002).

**Figure S5.** (A) Manhattan and (B) Q–Q plots for the genome‐wide time‐to‐event analysis of tolerance (adjusted for sex) (*λ *= 1.027).

**Figure S6.** Regional plot of the 11p13 locus rs11031684 identified in the genome‐wide time‐to‐event analysis of tolerance (data from analysis adjusted for sex).

**Figure S7.** (A) Manhattan and (B) Q–Q plots for genome‐wide time‐to‐event analysis of tolerance (adjusted for sex, age of daily smoking, CPD, and max CPD) (*λ *= 1.078).

**Figure S8.** Regional plot of the 9q34.12 locus rs2304808 identified in the genome‐wide time‐to‐event analysis of tolerance (data from analysis adjusted for sex, age of daily smoking, CPD, and max CPD).

**Figure S9.** Manhattan and Q–Q plots for the genome‐wide time‐to‐event analysis of cessation (adjusted for sex) (*λ *= 1.001).

**Figure S10.** Manhattan and Q–Q plots for the genome‐wide time‐to‐event analysis of cessation (adjusted for sex, FTND, and DSM‐IV nicotine withdrawal) (*λ *= 1.011).

**Figure S11.** Regional plot of the 10p14 locus rs72779075 identified in the genome‐wide time‐to‐event analysis of cessation (data from analysis adjusted for sex).

**Table S1.** Description of the NAG‐OZALC sample used for replication.

**Table S2.** Results of the replication study for SNPs located in *TRPM4* and *SLC6A16* (with ±50 kb flanking regions). For comparison, corresponding results from the Finnish discovery sample are also shown.

**Table S3.** List of SNPs in smoking‐related genes reported from previous GWAS that show nominal association (*P* < 0.05) with the transitions.

**Appendix S1.** Details of the mediation analyses.
