# Supplementary figures and images for: Genome‐wide time‐to‐event analysis on smoking progression stages in a family‐based study
Source: Brain Behav. 2016 Apr 22;6(5):e00462. doi: 10.1002/brb3.462 (PMC4842934; doi:10.1002/brb3.462)

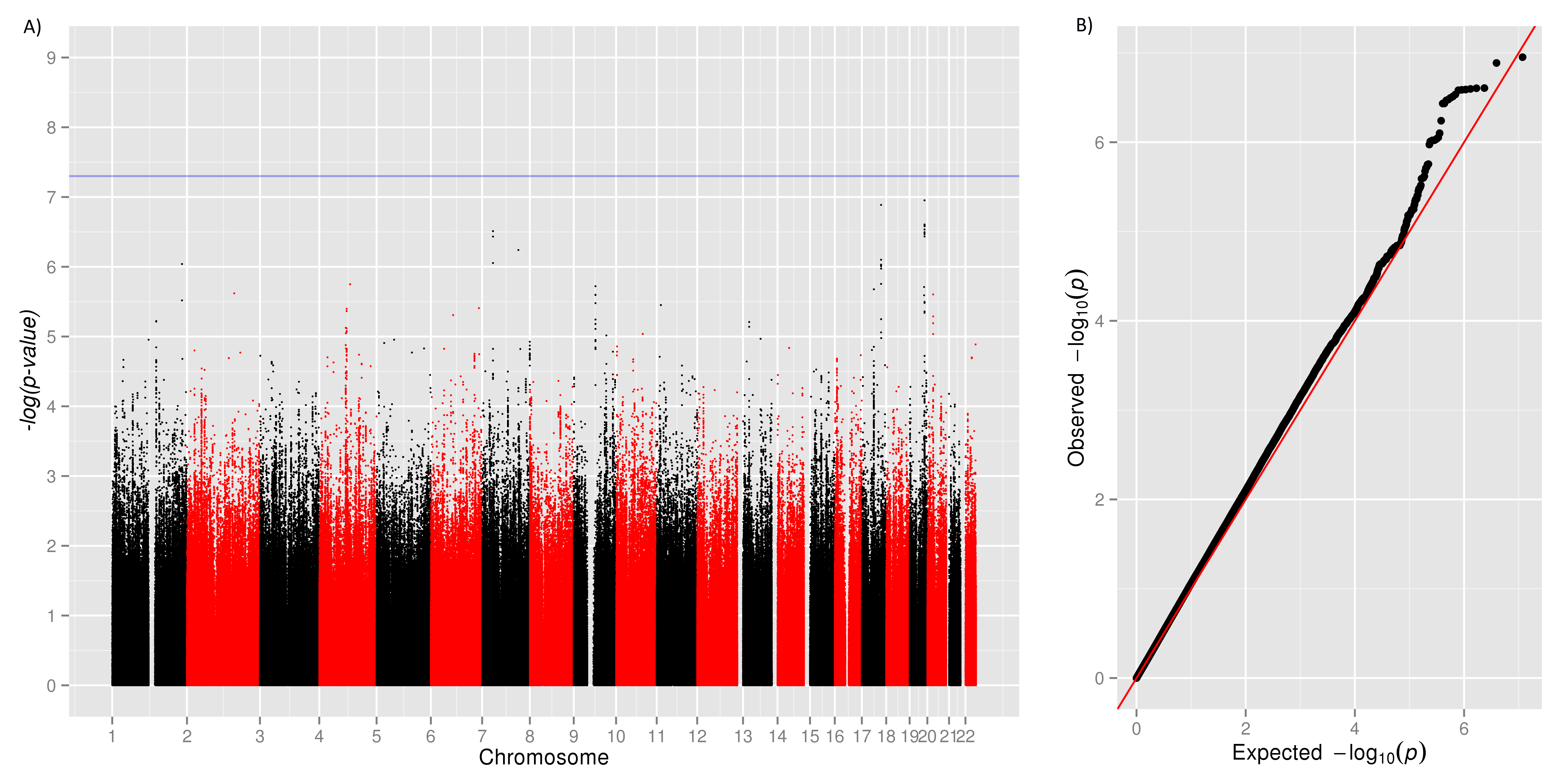

Supplement: Supplementary file 1 — Figure S1. (A) Manhattan and (B) Q–Q plots for the genome‐wide time‐to‐event analysis of smoking initiation (adjusted for sex and birth year) (λ = 1.083). [file BRB3-6-e00462-s001.tif]

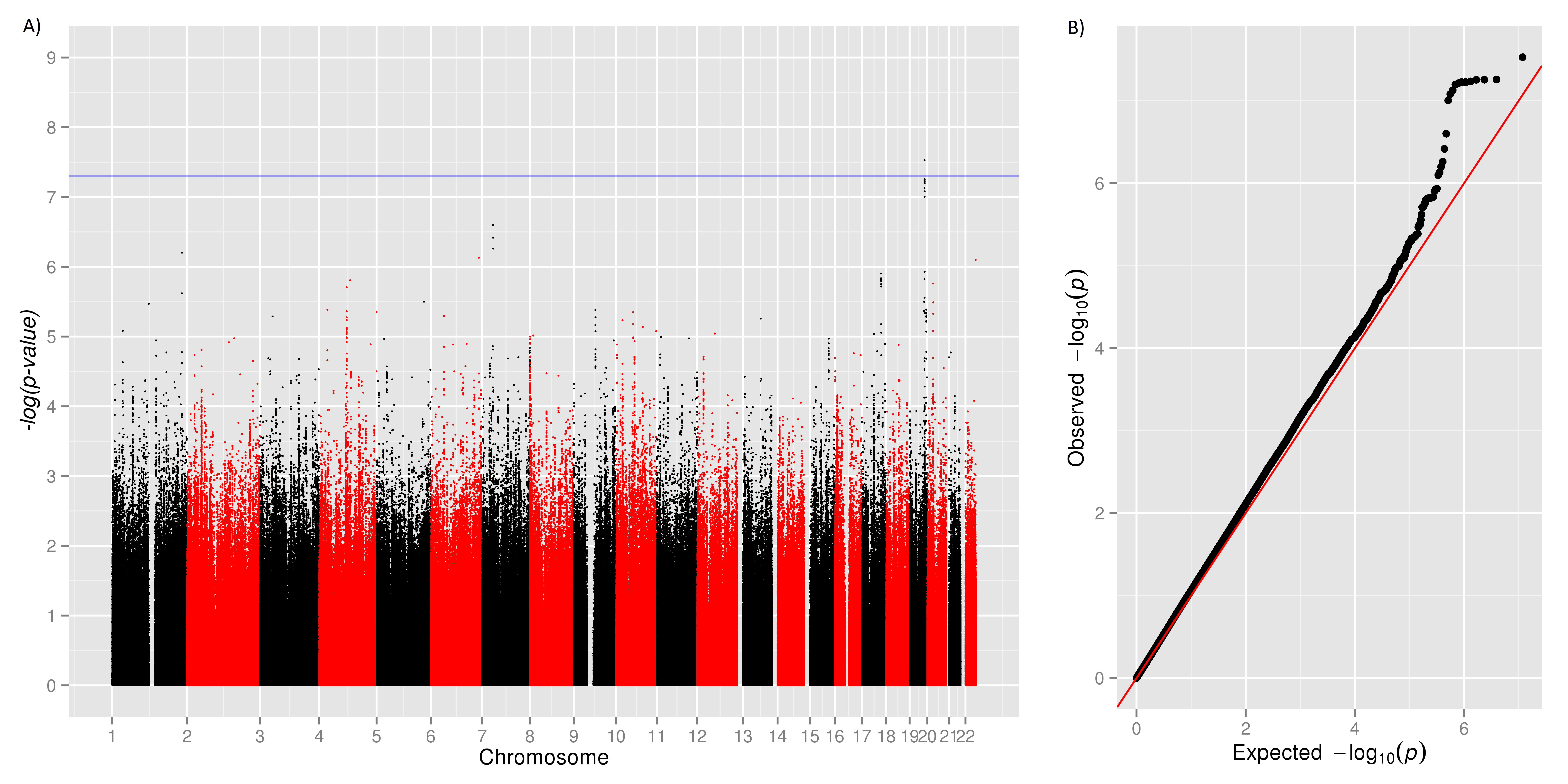

Supplement: Supplementary file 2 — Figure S2. (A) Manhattan and (B) Q–Q plots for the follow‐up analysis of smoking initiation (adjusted for sex and birth year, as well as positive and negative sensation scores) (λ = 1.089). [file BRB3-6-e00462-s002.tif]

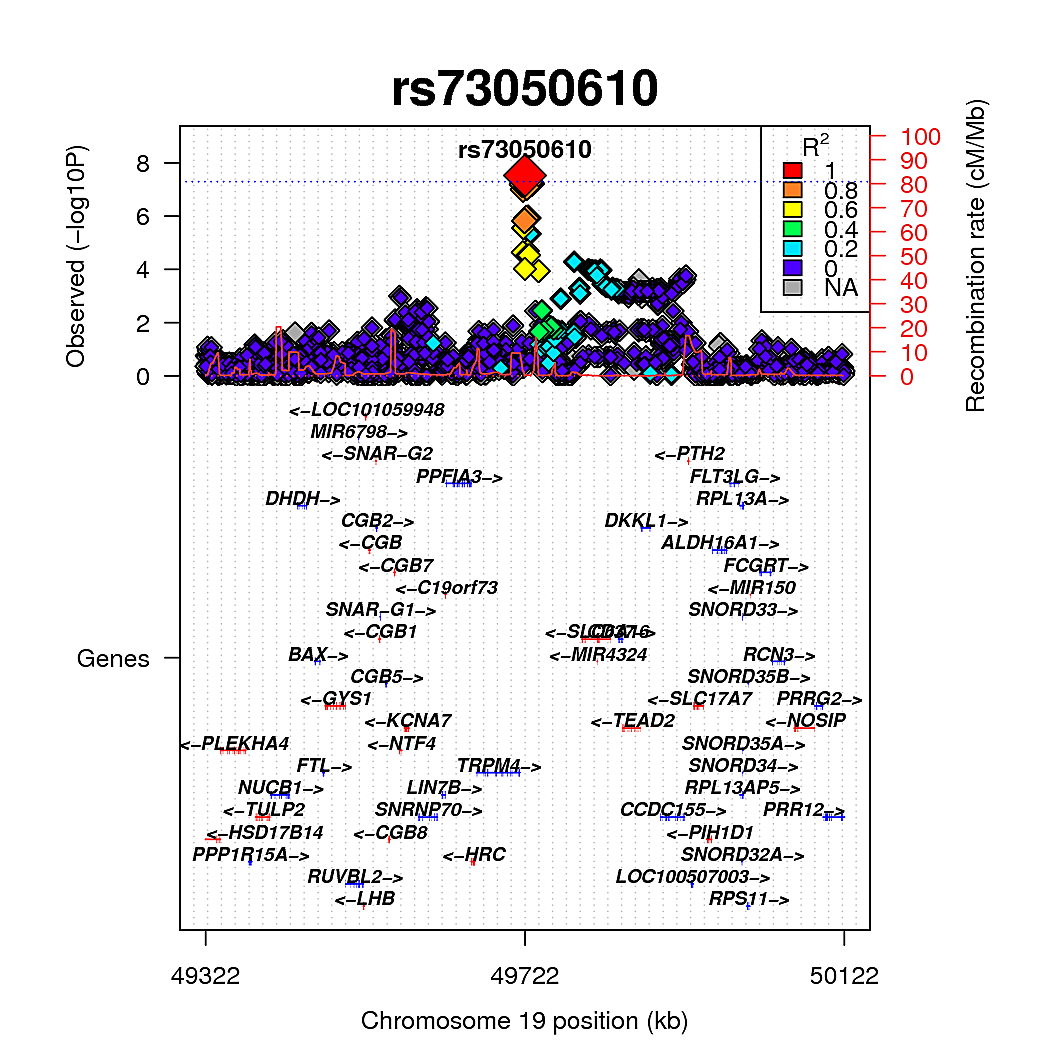

Supplement: Supplementary file 3 — Figure S3. Regional plot of the 19q13.33 locus rs73050610 identified for smoking initiation (data from analysis adjusted for sex and birth year, as well as positive and negative sensation scores). [file BRB3-6-e00462-s003.tif]

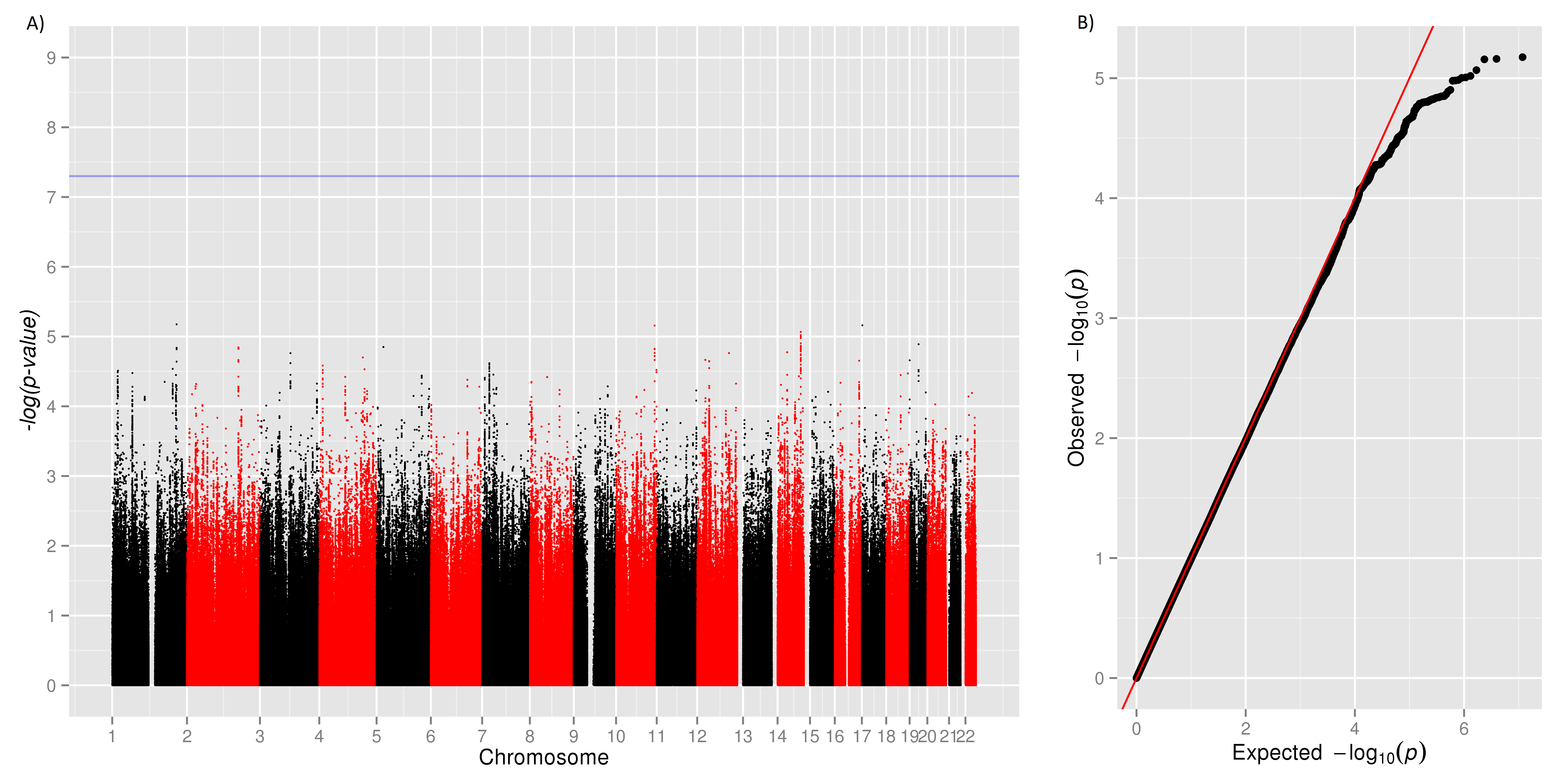

Supplement: Supplementary file 4 — Figure S4. (A) Manhattan and (B) Q–‐Q plots for the genome‐wide time‐to‐event analysis of persistent smoking (adjusted for sex) (λ = 1.002). [file BRB3-6-e00462-s004.tif]

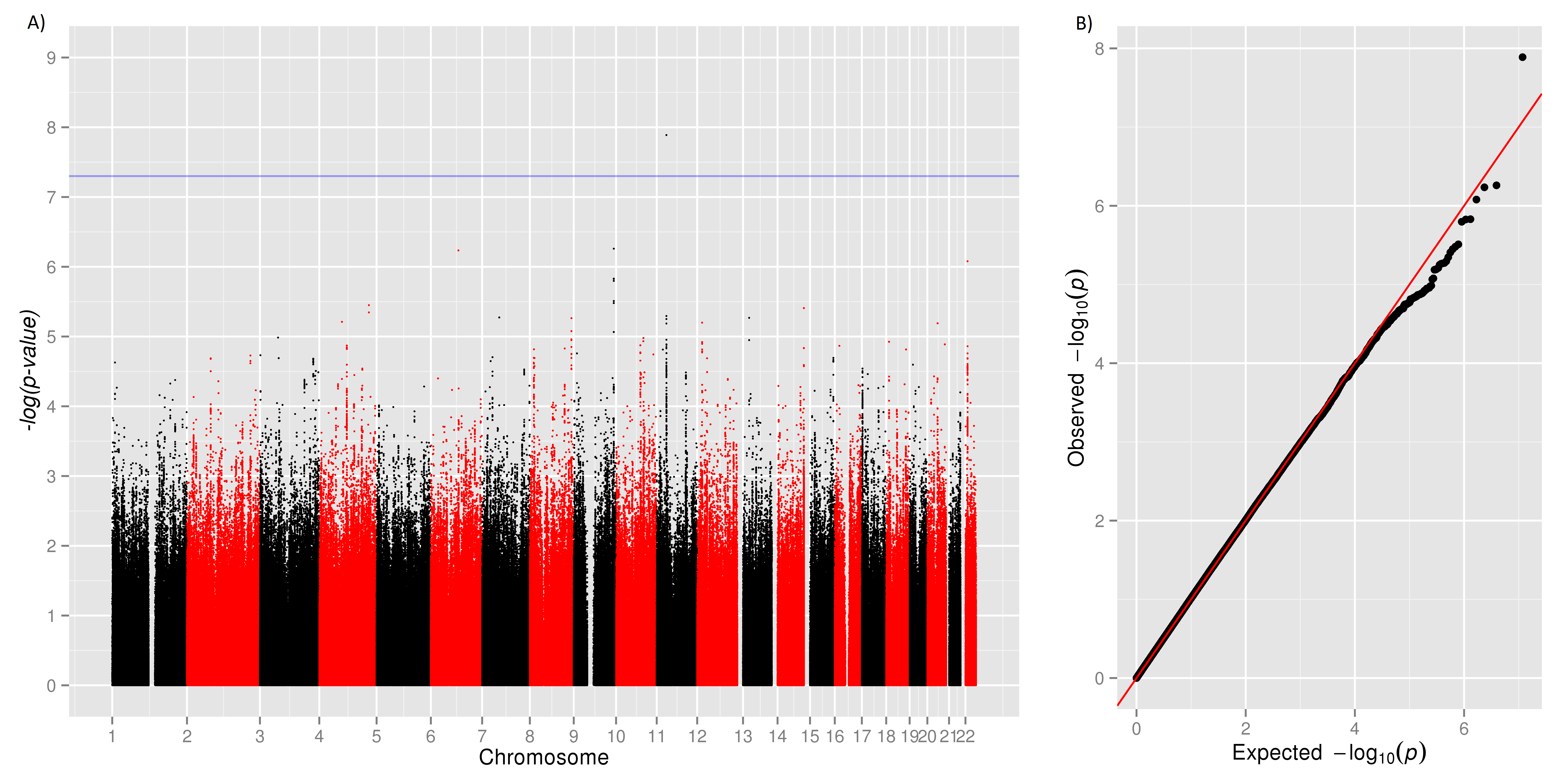

Supplement: Supplementary file 5 — Figure S5. (A) Manhattan and (B) Q–Q plots for the genome‐wide time‐to‐event analysis of tolerance (adjusted for sex) (λ = 1.027). [file BRB3-6-e00462-s005.tif]

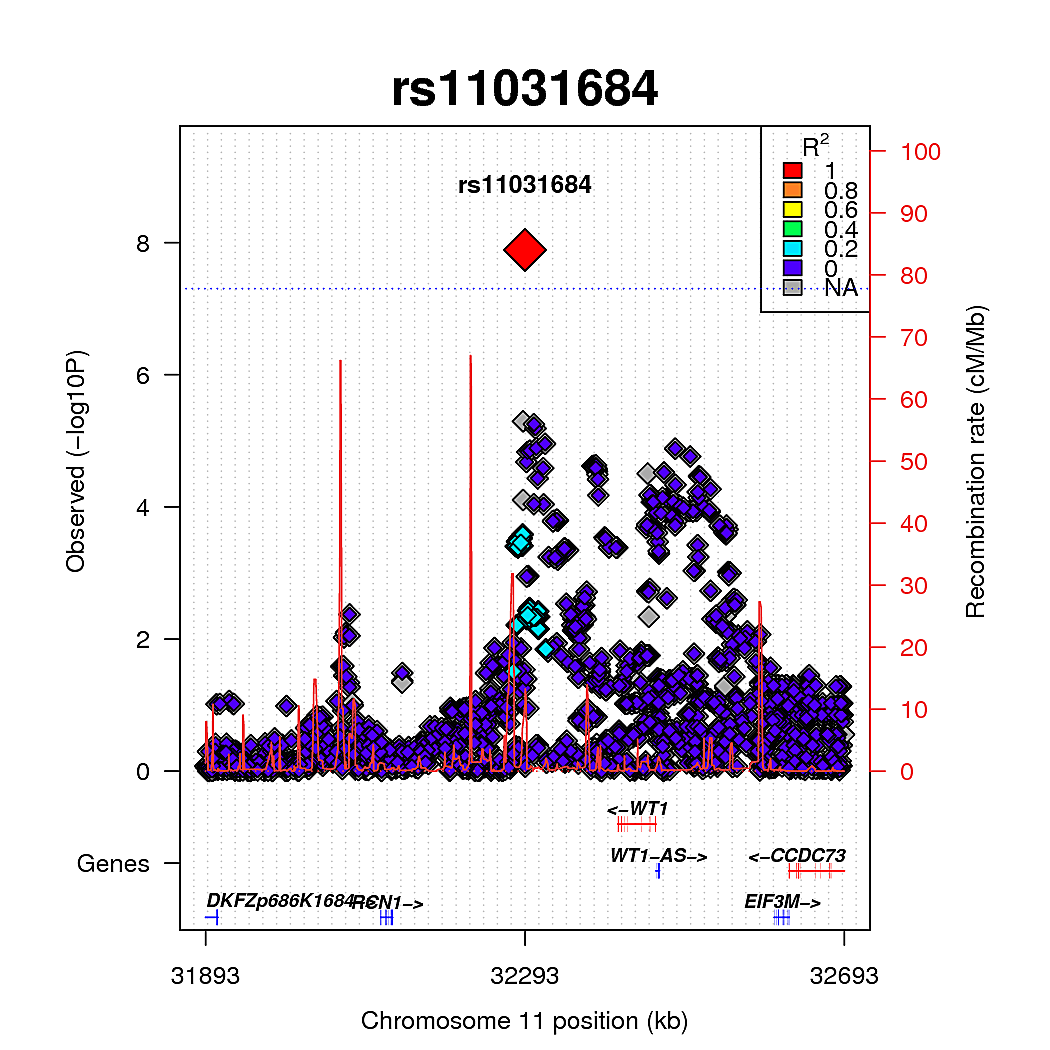

Supplement: Supplementary file 6 — Figure S6. Regional plot of the 11p13 locus rs11031684 identified in the genome‐wide time‐to‐event analysis of tolerance (data from analysis adjusted for sex). [file BRB3-6-e00462-s006.tif]

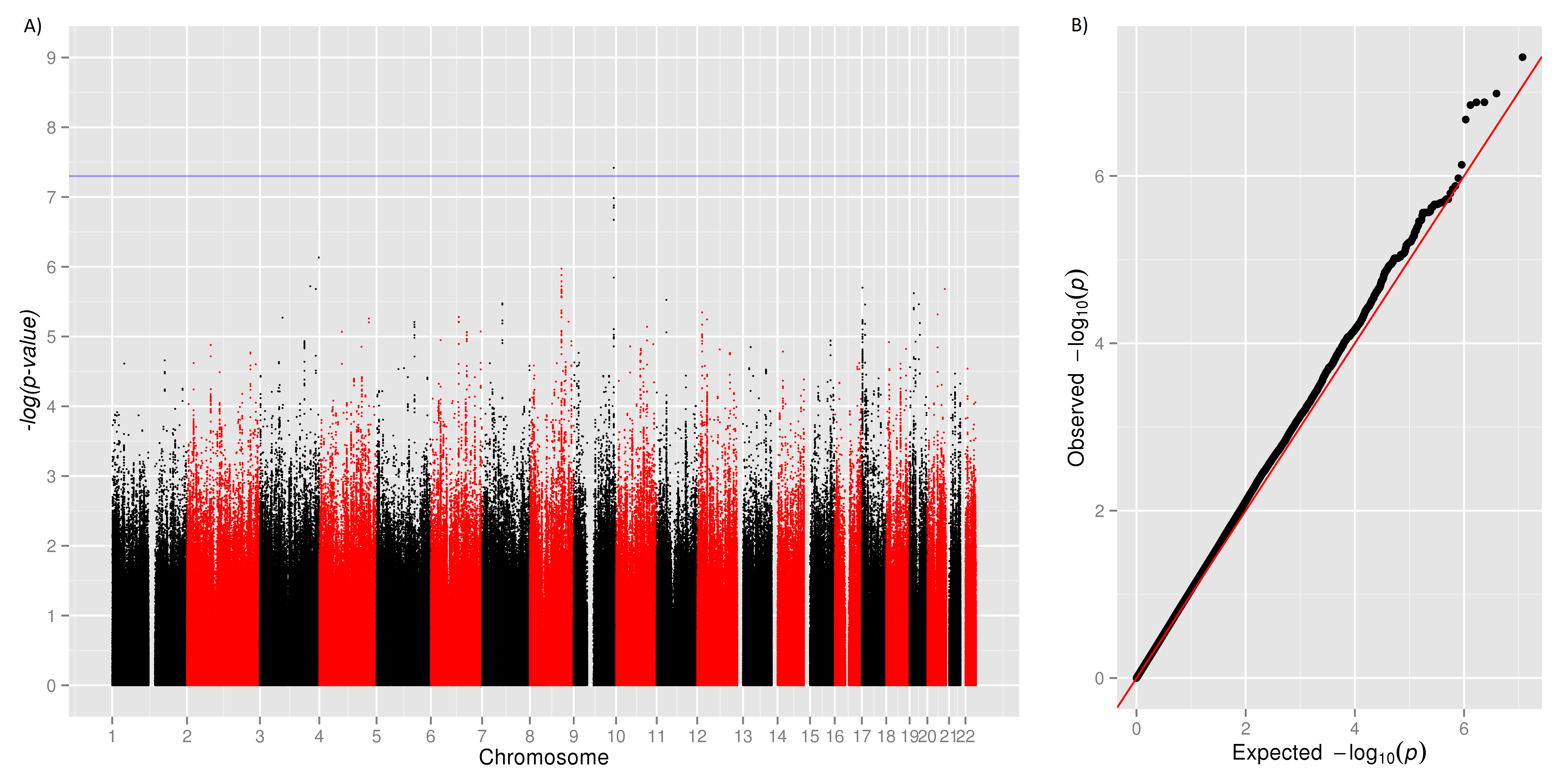

Supplement: Supplementary file 7 — Figure S7. (A) Manhattan and (B) Q–Q plots for genome‐wide time‐to‐event analysis of tolerance (adjusted for sex, age of daily smoking, CPD, and max CPD) (λ = 1.078). [file BRB3-6-e00462-s007.tif]

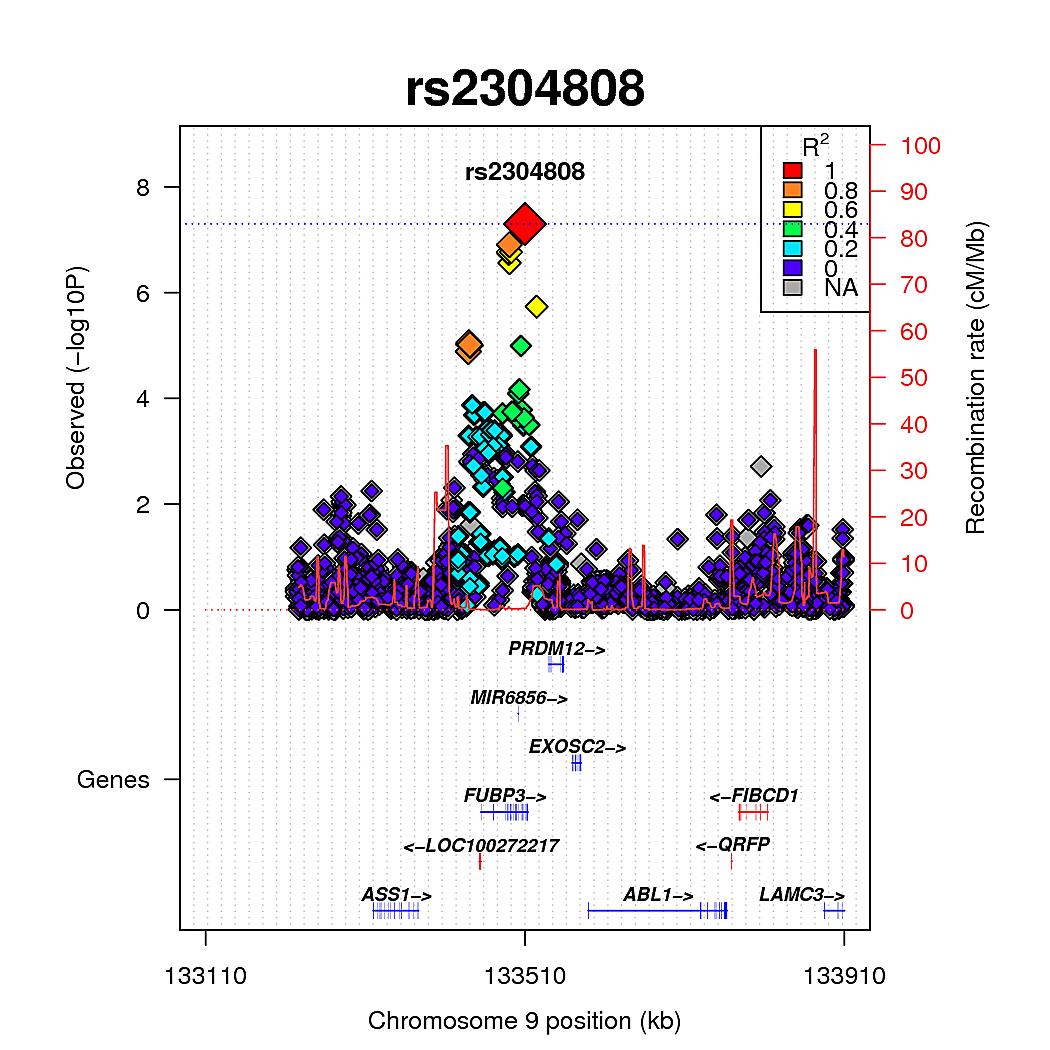

Supplement: Supplementary file 8 — Figure S8. Regional plot of the 9q34.12 locus rs2304808 identified in the genome‐wide time‐to‐event analysis of tolerance (data from analysis adjusted for sex, age of daily smoking, CPD, and max CPD). [file BRB3-6-e00462-s008.tif]

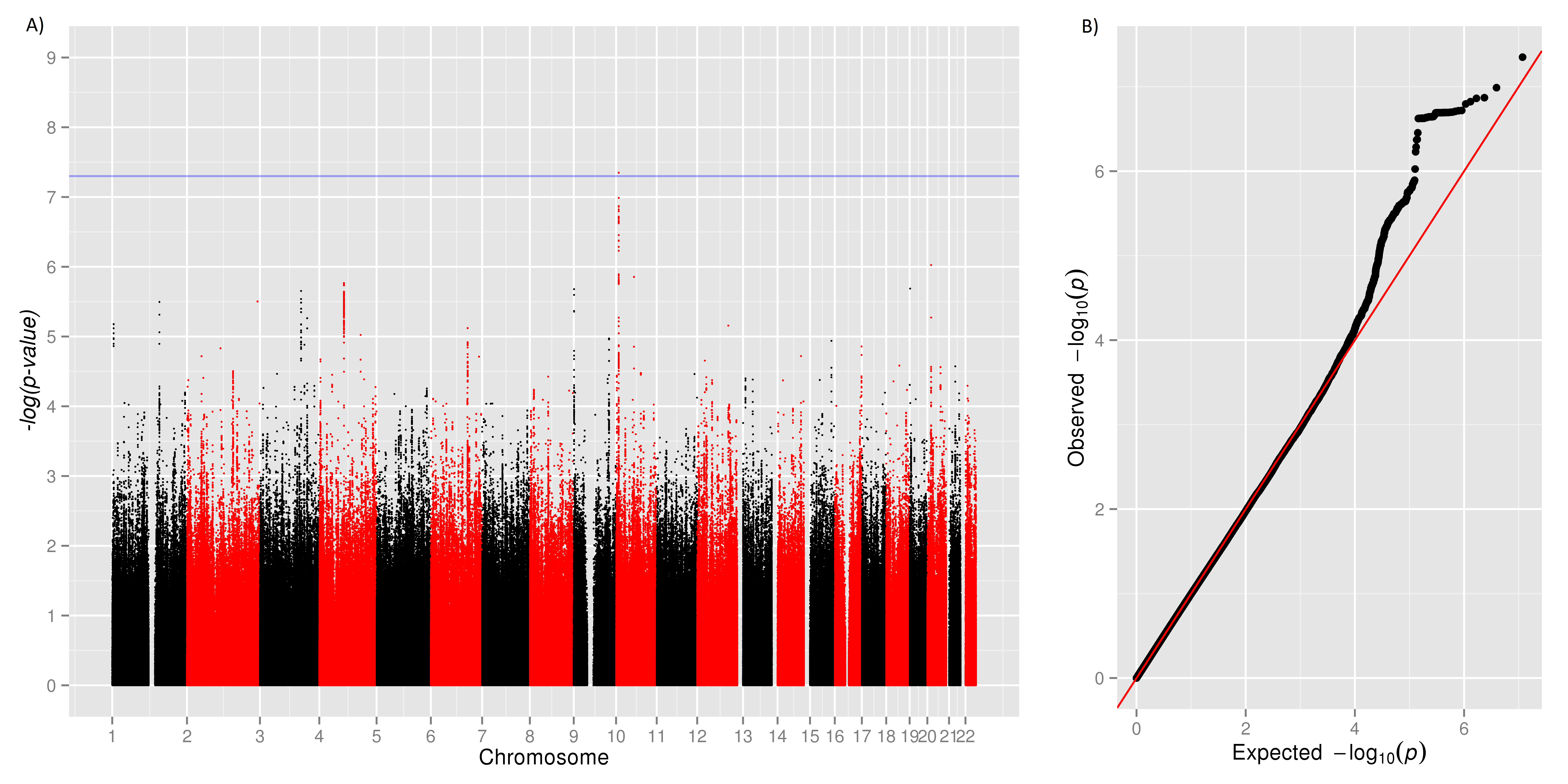

Supplement: Supplementary file 9 — Figure S9. Manhattan and Q–Q plots for the genome‐wide time‐to‐event analysis of cessation (adjusted for sex) (λ = 1.001). [file BRB3-6-e00462-s009.tif]

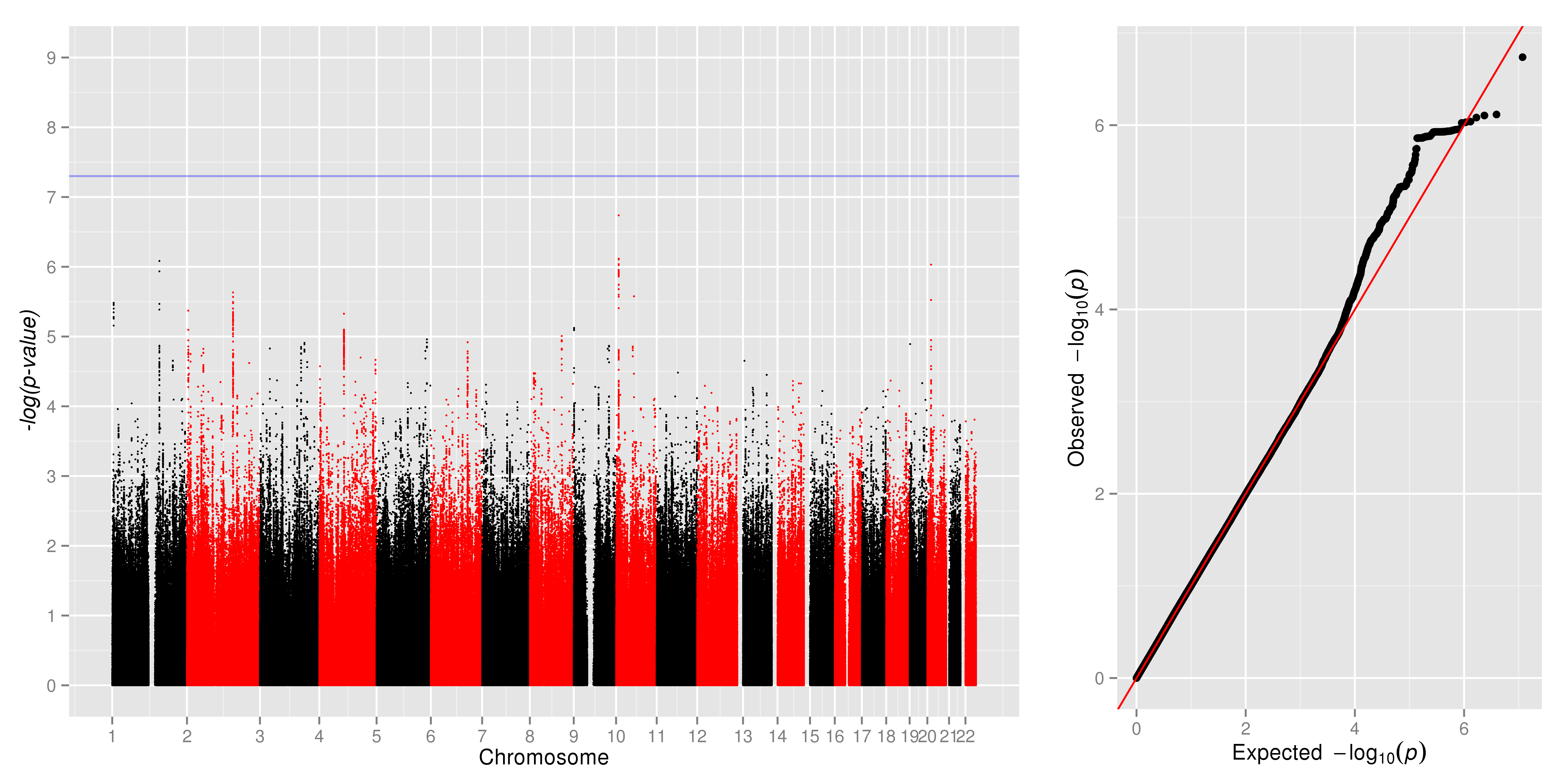

Supplement: Supplementary file 10 — Figure S10. Manhattan and Q–Q plots for the genome‐wide time‐to‐event analysis of cessation (adjusted for sex, FTND, and DSM‐IV nicotine withdrawal) (λ = 1.011). [file BRB3-6-e00462-s010.tif]

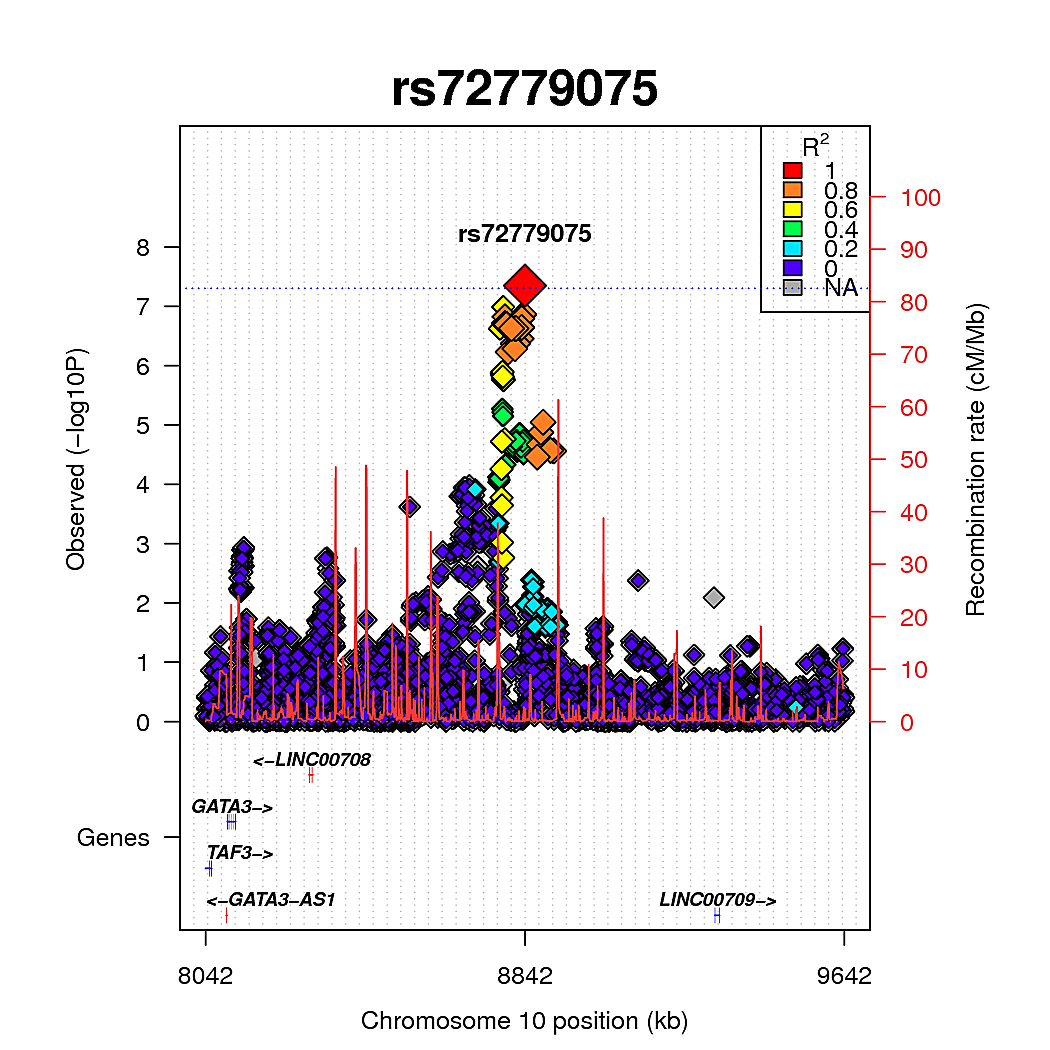

Supplement: Supplementary file 11 — Figure S11. Regional plot of the 10p14 locus rs72779075 identified in the genome‐wide time‐to‐event analysis of cessation (data from analysis adjusted for sex). [file BRB3-6-e00462-s011.tif]
