# Supplementary material for: Genome‐wide time‐to‐event analysis on smoking progression stages in a family‐based study
Source: Brain Behav. 2016 Apr 22;6(5):e00462. doi: 10.1002/brb3.462 (PMC4842934; doi:10.1002/brb3.462)
Supplement: Supplementary file 15 — Appendix S1. Details of the mediation analyses. [file BRB3-6-e00462-s015.docx]

# Supplemental text: Details of the mediation analyses

The mediation analyses were performed for the top significantly identified SNPs in three of the transitions of interest. Previous studies on the mediation analysis in the context of survival model indicate that the product method only produces a consistent estimate of mediation effect in the additive model but not the cox model although it can still be used to test the effect (VanderWeele 2011). In this study, we adopted the method proposed by Imai et al. and the corresponding R package ‘mediation’ (Imai *et al.* 2010; Tingley *et al.* 2014) to estimate the average causal mediation effect (ACME) because it provides a broader and more general framework to estimate and test mediation effects under various scenarios by utilizing Monte Carlo simulation. It should be noted that one of the limitations of the mediation analysis was that, although we included the covariates such as sex, it was still possible that there exist unmeasured confounders between the mediator and the outcome that was affected by a SNP. In this case, the mediation effect was not identifiable.

The mediation analysis for each SNP required specifying a mediation model and an outcome model. In the mediation model, we used linear (the ‘lm’ function) and ordered (the ‘polr’ function) logistic regression models for continuous (e.g. first time sensation scores) and ordinal (e.g. FTND, HSI) mediators, respectively. In the outcome model, we used a parametric survival model with a Weibull distribution (the ‘survreg’ function). Note that the interpretation of coefficients in survreg is different from those in coxme. Therefore, the estimated ACME cannot be directly compared with HRs in GWAS. Nevertheless, the coefficient from the Cox model is close to the negative product of that from survreg times the inverted scale estimate. Unfortunately, since the survival model with mixed effects has not been supported in the ’mediation’ R package, we included only independent individuals (n=691) in the mediation analyses, which might reduce the statistical power to detect the ACME. All estimates were obtained based on 1,000 replications.

# Reference

Imai K., Keele L., Tingley D., 2010 A general approach to causal mediation analysis. Psychol. Methods **15**: 309–334.

Tingley D., Yamamoto T., Hirose K., Keele L., Imai K., 2014 Mediation: R package for causal mediation analysis. J. Stat. Softw. **59**.

VanderWeele T. J., 2011 Causal mediation analysis with survival data. Epidemiol. Camb. Mass **22**: 582–585.
